# Effects of Mindful Eating in Patients with Obesity and Binge Eating Disorder

**DOI:** 10.3390/nu16060884

**Published:** 2024-03-19

**Authors:** Tatiana Palotta Minari, Gerardo Maria de Araújo-Filho, Lúcia Helena Bonalume Tácito, Louise Buonalumi Tácito Yugar, Tatiane de Azevedo Rubio, Antônio Carlos Pires, José Fernando Vilela-Martin, Luciana Neves Cosenso-Martin, André Fattori, Juan Carlos Yugar-Toledo, Heitor Moreno

**Affiliations:** 1 Department of Psychiatry, State Faculty of Medicine of São José do Rio Preto (FAMERP), São José do Rio Preto 15090-000, SP, Brazil; 2 Department of Hypertension, State Faculty of Medicine of São José do Rio Preto (FAMERP), São José do Rio Preto 15090-000, SP, Brazil; 3 Department of Endocrinology, State Faculty of Medicine of São José do Rio Preto (FAMERP), São José do Rio Preto 15090-000, SP, Brazil; 4 School of Medical Sciences, State University of Campinas (UNICAMP), Campinas 13083-887, SP, Brazil; 5 Cardiovascular Pharmacology & Hypertension Laboratory, School of Medical Sciences, State University of Campinas (UNICAMP), Campinas 13083-887, SP, Brazil

**Keywords:** eating disorders, binge eating disorder, obesity, nutritional intervention, mindful eating

## Abstract

Introduction: Binge eating disorder (BED) is a psychiatric illness related to a high frequency of episodes of binge eating, loss of control, body image dissatisfaction, and suffering caused by overeating. It is estimated that 30% of patients with BED are affected by obesity. “Mindful eating” (ME) is a promising new eating technique that can improve self-control and good food choices, helping to increase awareness about the triggers of binge eating episodes and intuitive eating training. Objectives: To analyze the impact of ME on episodes of binge eating, body image dissatisfaction, quality of life, eating habits, and anthropometric data [weight, Body Mass Index (BMI), and waist circumference] in patients with obesity and BED. Method: This quantitative, prospective, longitudinal, and experimental study recruited 82 patients diagnosed with obesity and BED. The intervention was divided into eight individual weekly meetings, guided by ME sessions, nutritional educational dynamics, cooking workshops, food sensory analyses, and applications of questionnaires [Body Shape Questionnaire (BSQ); Binge Eating Scale (BES); Quality of Life Scale (WHOQOL-BREF)]. There was no dietary prescription for calories, carbohydrates, proteins, fats, and fiber. Patients were only encouraged to consume fewer ultra-processed foods and more natural and minimally processed foods. The meetings occurred from October to November 2023. Statistical analysis: To carry out inferential statistics, the Shapiro–Wilk test was used to verify the normality of variable distribution. All variables were identified as non-normal distribution and were compared between the first and the eighth week using a two-tailed Wilcoxon test. Non-Gaussian data were represented by median ± interquartile range (IQR). Additionally, α < 0.05 and *p* < 0.05 were adopted. Results: Significant reductions were found from the first to the eighth week for weight, BMI, waist circumference, episodes of binge eating, BSQ scale score, BES score, and total energy value (all *p* < 0.0001). In contrast, there was a significant increase in the WHOQOL-BREF score and daily water intake (*p* < 0.0001). Conclusions: ME improved anthropometric data, episodes of binge eating, body image dissatisfaction, eating habits, and quality of life in participants with obesity and BED in the short-term. However, an extension of the project will be necessary to analyze the impact of the intervention in the long-term.

## 1. Introduction

Binge eating disorder (BED) is a psychiatric illness that occurs in approximately 3% of the population. The diagnosis comprises a high frequency of binge eating episodes, suffering caused by excessive eating, and dissatisfaction with body image. It is estimated that 30% of these patients are affected by obesity [1]. Binge eating episodes are characterized by a loss of control over eating and a high food intake, far beyond what that person would normally eat. They usually occur at 2 h intervals and can reach an intake from 2000 kcal to 5000 kcal per episode [2]. The patient suffers from abdominal pain, guilt, shame, and impulsivity, but this behavior is not associated with subsequent compensatory/purgatory actions [1]. According to the literature, BED can be triggered by emotional factors, which are closely linked to family relationships, personal conflicts, severe dietary restrictions, dissatisfaction with body image, boredom, and media [3].

Parallel to this scenario, global obesity rates have almost tripled in the last 50 years [4]. Overweight is a major public health concern and has been associated with a higher prevalence of psychiatric and chronic non-communicable diseases [4,5]. New approaches to prevent and treat obesity [5] which seek to reduce the emphasis on weight loss as the main health objective and try to reduce the stigma towards people who are overweight have been increasing [6]. This is because traditional interventions focused heavily on weight loss have not produced good long-term results and weight regain is often observed [7]. Furthermore, obesity is a chronic and recurrent disease [4]. In other words, there is no “cure”, just “control”, which means that there is no clinically effective method that can promise “definitive weight loss” [7]. After all, the body’s natural tendency is recovery [5]. Therefore, long-term maintenance does not depend on how you lost the weight (or even how quickly), but rather what you will do after having lost it and also what you will continue to do [5,7].

In recent years, the number of patients with obesity who are also affected by BED has grown [3,8]. Unfortunately, there are not many studies which combine treatments for both diseases [9,10]. The “Mindfulness for Eating Program”, also called “mindful eating” (ME), provides new and strong evidence for the treatment of people who are overweight and also have eating disorders [11]. This therapy integrates the practice of mindfulness with the science of nutrition, psychology, body awareness, and self-control [12]. Studies have demonstrated significant improvement of dietary regulation. ME can be a great alternative to severely restrictive diets which tirelessly seek to meet the social norms of an unattainable body standard [8,13,14].

Traditionally, ME is defined as the act of eating consciously, maintaining full attention in the moment, and letting go of judgments about one’s own eating practices [12]. With breathing control, the patient gently slows down racing thoughts, focusing only on their body and food [11]. It is not a diet or a list of “forbidden” or “allowed” foods, as it is not just about “what you should eat?”. The practice teaches “how and why we eat?” [14]. The technique is also made up of four elements: “savoring”, “observing”, “non-judgmental”, and “in the moment” [12]. In general, the patient is encouraged to turn off the TV, radio, and cell phone during meals; use their non-dominant hand to pick up the fork; chew their food well before swallowing it; feel the texture, colors, and flavors of the food; control their breathing to reduce anxiety before eating; enjoy their meal without any judgment; identify the physiological signs of hunger and satiety; eat calmly and without rushing [12,13,14].

Unfortunately, food has become a source of suffering for many people, mainly patients with obesity and BED, who feel fear, guilt, or anxiety before, during, and after meals [6]. These emotions are the result of nutritional terrorism, a phenomenon marked by severe caloric restriction and psychological pressure to follow them [11]. Therefore, ME helps increase awareness of the triggering factors involved in compulsion (physiological, environmental, or emotional factors) and intuitive eating training (respecting the signs of hunger and satiety) [14], and not only emphasizes the amount ingested, but also the use of the experience of eating, thus changing the value of the food reward without restricting it [12].

According to some research, individualized management and constant communication with a specialized multidisciplinary team (psychiatrist, nutritionist, psychologist, and physical educator) are also a part of the treatment of various diseases [14], especially for people who are overweight and also have eating disorders [15,16,17]. Nutritionists can prioritize some techniques that can approach food supply in a qualitative way, showing that nutrition can go far beyond the prescription of macro- and micronutrients [16,18]. In addition, patients also learn to identify the causes of binge eating episodes, applying some techniques that can control it, such as a food diary [16,18] and also ME [12].

A recent randomized clinical trial showed that ME appears to be effective in reducing emotional eating behavior, increasing the quality of food intake, and developing self-compassion in people who are overweight. However, these good results were not sustained in terms of weight loss, thus increasing the uncertainty regarding this topic [5]. In this sense, more studies are needed that can particularly illustrate the application of ME in patients with obesity who also have BED [8].

Furthermore, ME refers to a diverse set of practices that can have very different effects on behavior. Another problem would be the ways in which ME is performed and measured, as well as its effects and potential mechanisms of action [19,20]. Interventions based on multiple components, including ME, may be beneficial for eating disorders and weight loss, but it is unclear whether these benefits are unique to ME or underlie other added therapies [19,20]. The literature also suggests that ME may have immediate effects on nutrition, but these effects may vary depending on the individual and dietary context, suggesting further studies with different types of patients and diseases [20].

Given the lack of literature on effective treatments for controlling BED and obesity, the low number of studies on the application of ME in both diseases, and the various limitations found in research on ME, the main interest in carrying out this work was driven by the intention to promote an innovative nutritional intervention that can address more subjective aspects of the management of this public health concern. Therefore, the objective of this study was to analyze the impact of ME on episodes of binge eating, body image dissatisfaction, quality of life, eating habits, and anthropometric data [weight, Body Mass Index (BMI), and waist circumference] in patients with obesity and BED during an eight-week nutritional intervention.

## 2. Materials and Methods

### 2.1. Ethical Aspects

This study was conducted in accordance with the Declaration of Helsinki and approved by the Institutional Ethics Committee of the State Faculty of Medicine in São José do Rio Preto (FAMERP), São José do Rio Preto—São Paulo, Brazil—Human Research Ethics Committee (CAAE: 70217317.4.0000.5415), with first approval on 18 July 2017. It was also registered in Clinical Trials (NCT06230107). Informed consent was obtained from all subjects involved in this study and all patients agreed to participate of their own free will. Written informed consent was also obtained from patients to publish this paper. Confidentiality and anonymity regarding content were guaranteed in order to preserve the identity of the interviewees. The study data were collected and managed using REDCap 14.0.9 electronic data capture tools hosted at REDCap—FUNFARME/FAMERP, State Faculty of Medicine of São José do Rio Preto—São José do Rio Preto, São Paulo, Brazil (https://redcap.hospitaldebase.com.br:44312/, accessed from October to December 2023) [21,22].

### 2.2. Study Design

In this prospective, longitudinal, and experimental study, participants underwent a nutritional intervention with the practice of ME for 8 weeks. Recruitment took place through a flyer posted on social media, and patients from previous studies who were interested in participating were also invited. Meetings were held at the Nutritional Clinic (São José do Rio Preto—SP/Brazil) and took place from October to November 2023. The consultation was scheduled individually, lasting 1 h. Around 13 to 14 patients were seen per day of the week, during 6 working days, by members of the research team. The clinic’s daily opening hours were from 8 am to 6 pm, Monday to Friday, and 8 am to 12 pm on Saturdays. All patients were seen once a week (Monday to Saturday) during the 2 months of intervention. This study began in early October and lasted until mid-November.

Before each meeting, the researchers contacted the participants reminding them of the appointment. After the intervention, another telephone contact was also made with the patients, inviting them to participate in a follow-up. The objective of this last contact was to analyze the stability of behavior after the study. The follow-up took place 8 weeks after the end of the intervention (second week of January).

### 2.3. Participants

**Figure 1:**
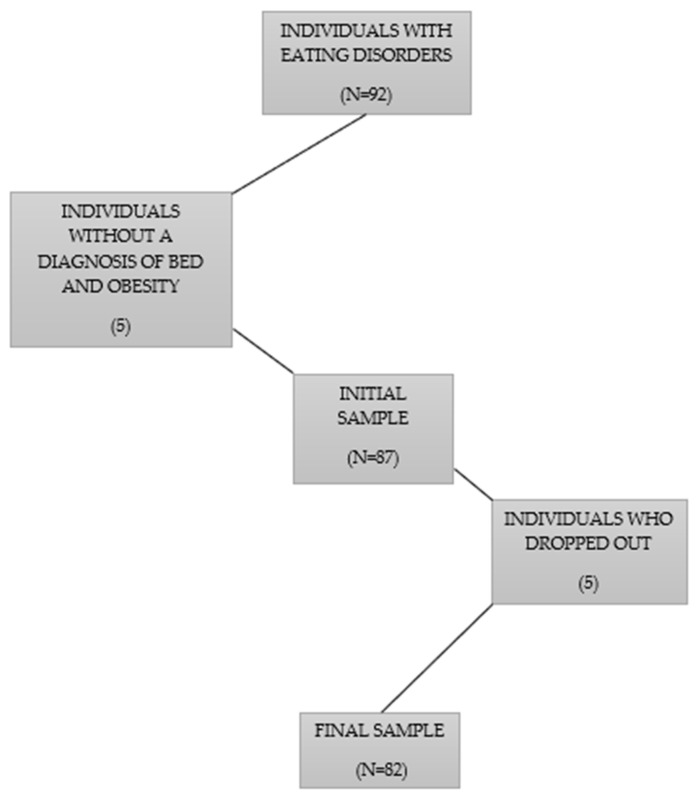
Flowchart of the sample selection.

This study recruited patients diagnosed with obesity and BED. The inclusion criteria were age ≥18 years; diagnosis of obesity (BMI ≥ 30 kg/m³) and BED (according to DSM-5 [1]); willingness to participate in the intervention. The exclusion criteria were patients with clinical or cognitive impairments affecting their response to the instruments and individuals without a diagnosis of obesity and BED. At the end of the screening, 82 patients were selected. The flowchart of the sample selection is represented in Figure 1.

All patients were monitored by a psychiatrist and psychologist (cognitive–behavioral therapy). They also were sedentary and did not take any continuous medication.

### 2.4. Study Protocol

The eight meetings were based on Brazilian nutritional education dynamics that are carried out in the “Dietary Guidelines for the Brazilian Population—Ministry of Health/Brazil” [23] and in the “Integral Nutritional Care Program—Mindful Eating” [12]. There was no dietary prescription for calories, carbohydrates, proteins, fats, and fiber. Patients were only encouraged to consume fewer ultra-processed foods and more natural and minimally processed foods. They were instructed to use ME during their daily meals and were also encouraged to bring one of their favorite foods to practice the technique at all meetings. Table 1 summarizes the meetings, goals, and procedures during the 8 weeks of intervention.

### 2.5. Instruments

Details of the protocols and questionnaires that were applied are as follows:

1. Clinical and sociodemographic protocol: collects general clinical assessment information (signs, symptoms, disease, family history, dietary habits, and water intake), socioeconomic level, race, sex, age, occupation, educational degree, and other general information [24];
2. “Quality of Life Scale WHOQOL-Bref”: evaluates the patient’s quality of life and consists of 26 questions on a Likert scale. This questionnaire is divided into 4 domains—physical, psychological, social, and environmental. According to the classification, a higher percentage indicates a better quality of life (range of scale values = minimum 0% and maximum 100%). The Cronbach’s alpha coefficient of the WHOQOL-BREF was 0.89. Internal reliability for all domains was above 0.70 [25]. This questionnaire was also translated into and validated in Brazilian Portuguese and its Cronbach’s alpha coefficient was 0.77 for the domains and 0.91 for the questions [26];
3. “Body Shape Questionnaire (BSQ)”: measures satisfaction and concern with body shape, consisting of 34 items on a 6-point Likert scale. According to the classification, to be classified as “satisfied with your body shape”, a score <111 is necessary (range of scale values = minimum 34 points and maximum 204 points). Internal consistency, calculated in terms of Cronbach’s alpha, was 0.94 [27]. This questionnaire was also translated into and validated in Brazilian Portuguese and its Cronbach’s alpha coefficient was 0.97 [28];
4. “Binge Eating Scale (BES)”: quantifies the severity of binge eating, cognitions, feelings, and behaviors, consisting of 16 items in a 4-point Likert format and 62 statements that best represent the individual’s response. Each statement corresponds to a number of points from 0 to 3, ranging from the absence (“0”) to the maximum severity (“3”) of binge eating. The final score is the result of the sum of the points for each item. According to the classification, individuals with a score less than or equal to 17 are classified as “Without Binge Eating”. Individuals with a score between 18 and 26 are considered to have “Moderate Binge Eating”, and those with a score greater than or equal to 27 are classified as “Severe Binge Eating” (range of scale values = minimum 0 points and maximum 48 points). The internal consistency, calculated in terms of Cronbach’s alpha, was 0.89 [29]. This questionnaire was also translated into and validated in Brazilian Portuguese and its Cronbach’s alpha coefficient was 0.85 [30].
  Authorization and consent were obtained from all authors of the questionnaires and protocols that were applied in this study.

### 2.6. Data Collection, Considerations, and Additional Information

Weight (in kilograms/kg) and height (in centimeters/cm) were measured using “Digital Stainless-Steel Scale with 300 KG capacity—Brand Micheletti and Model MIC A300^®^, São Paulo/SP—Brazil” and “Sanny Scientific Stadiometer Model nº 30.25-481^®^, São Paulo/SP—Brazil”, respectively. Prior to the consultation, all patients were instructed to attend the meeting wearing light clothing and not having eaten large meals. For the measurements, they were asked to remove their shoes, jewelry, and empty their urinary bladder. The individuals were sequentially asked to step on the scale to measure their body weight. Meanwhile, to measure height, the patients were asked to position themselves on the stadiometer with their spine close to its rod, in an orthostatic posture (looking at the horizon and rib cage expanded). Data were always collected at the same time for each patient, aiming to mitigate any type of recurring body fluid fluctuation throughout the day [31].

Body Mass Index (BMI) (in kg/m^2^) calculation was expressed by the formula weight (in kg) divided by height squared (in m^2^), considering the following classification: underweight < 18.5 kg/m^2^; eutrophic or normal ≥ 18.5–24.9 kg/m^2^; overweight ≥ 25.0–29.9 kg/m^2^; obesity I ≥ 30.0–34.9 kg/m^2^; obesity II ≥ 35.0–39.9 kg/m^2^; obesity III ≥ 40 kg/m^2^ [32].

Waist circumference (in cm) was measured using the “2.0m Sanny^®^ Anthropometric Measuring Tape, São Paulo/SP—Brazil”. Before measuring, the midpoint between the lower rib and the hip bone was drawn on the patient’s body with a marker pencil designed for anthropometric measurements. Next, the tape was passed exactly through the midpoint, that is, in the waist circumference of each patient. Values below 80 cm for women and 94 cm for men indicate “low cardiovascular risk”. Values from 80 cm to 87 cm for women and 94 cm to 101 cm for men indicate “high cardiovascular risk”. Finally, values above 88 cm for women and 102 cm for men indicate “very high cardiovascular risk” [32].

To designate social class, the following classifications were considered: Class A (those earning more than 20 minimum wages); Class B (from 10 to 20 minimum wages); Class C (from 4 to 10 minimum wages); Class D (from 2 to 4 minimum wages); and Class E (receives up to 2 minimum wages). At the end of 2023, the value of a minimum wage was BRL 1320. (Note: BRL 1320 are equivalent to approximately USD 265 or EUR 244, considering the values of currencies that were quoted at the end of 2023) [33].

Total energy value (TEV) and water intake values were expressed in calories (Kcal) and liters (L), respectively, measured by participants’ food diary and also the “Clinical and Sociodemographic Protocol”.

### 2.7. Data Analysis

Initially, the variables “sex”, “race”, and “socioeconomic level” were converted into percentages relative to the total number of individuals who participated in the study. The variable “age” was represented by a descriptive range (minimum and maximum), as well the median ± interquartile range (IQR) (Md ± IQR), and additionally the mean ± standard deviation (SD) ($\bar{X}$ ± SD). All these data are included in a descriptive table that illustrates the sociodemographic data of the sample. 

Subsequently, to carry out inferential statistics, the Shapiro–Wilk test was conducted on the variables “weight”, “Body Mass Index (BMI)”, “waist circumference”, “Body Shape Questionnaire (BSQ)”, “Binge Eating Scale (BES)”, “number of binge eating episodes”, “Quality of Life Scale—WHOQOL BREF”, “total energy value (TEV)”, and “water intake”, observed in the first and eighth weeks, to verify normality of distribution [34].

After the Shapiro test, all variables were identified as non-normal distribution and were compared, between the 1st and 8th week, using the two-tailed Wilcoxon test. Non-Gaussian data are represented by median ± interquartile range (IQR).

TEV and water intake are represented in a descriptive table and other variables in graphs. Additionally, before each graph, there is an explanatory text which provides the exact values of each variable declared, where non-normal values are represented by median ± interquartile range (IQR). For curiosity, mean ± standard deviation (SD) values (represented by the symbol $\bar{X}$ ± SD) are also placed next to the median ± interquartile range (IQR).

All graphs (boxplots) show the mean (plus sign), the median (central boxplot line), the interquartile range (lower box limit for Q1 and upper box limit for Q3), the minimum (lower whisker), and the maximum (upper whisker).

A *p*-value ≥ 0.05 was adopted for the normality test and a *p*-value ≤ 0.05 was adopted for the other tests. Additionally, a significance level (α) ≤ 0.05 was adopted for all tests performed. All analyses and graph construction were performed using the GraphPad Prism 9.0 software [35].

## 3. Results

### 3.1. Sociodemographic Data

The majority of the participants were female, economic class C, of yellow race, and between 37 and 56 years old. Race, sex, and age were self-declared by all participants. Sociodemographic data are represented in Table 2.

### 3.2. Weight

**Figure 2:**
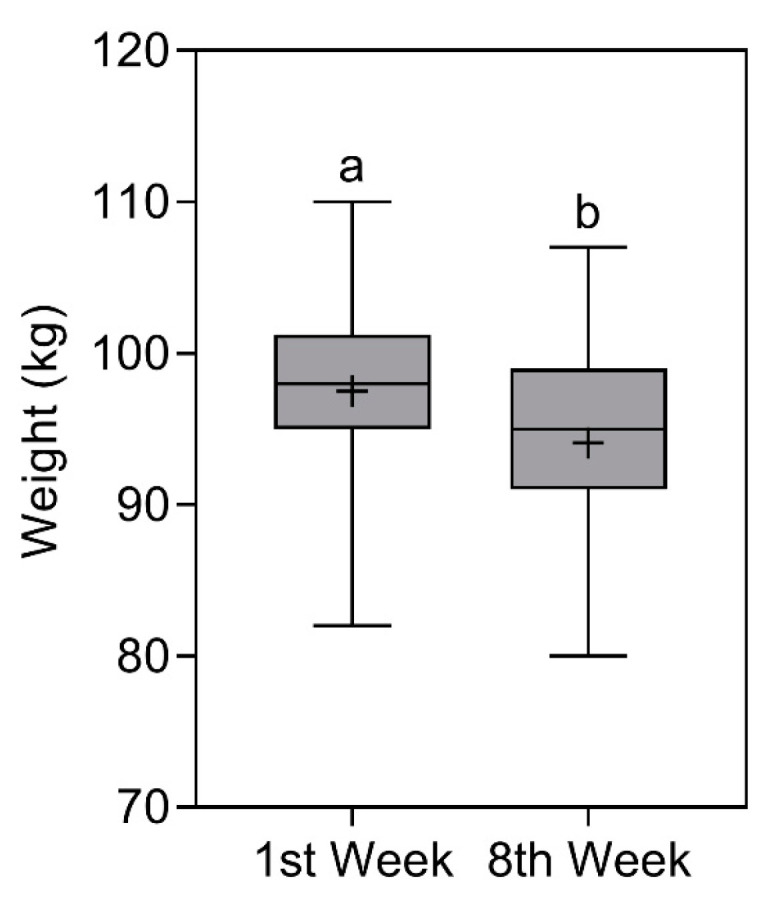
Body weight of study participants in the first and eighth week. Data presented as median (central boxplot line), mean (plus sign), interquartile range (lower box limit for Q1 and upper box limit for Q3), minimum (lower whisker), and maximum (upper whisker). Different superscript letters indicate a significant difference between the body weight observed in the first and eighth week (Wilcoxon test; *p* < 0.0001).

Weight reduced significantly (*p* < 0.0001) from 98 (101.3–95) kg [$\bar{X}$ ± SD = 97.5 ± 6.5 kg] in the first week to 95 (99–91) kg [$\bar{X}$ ± SD = 94.1 ± 6 kg] in the eighth week. The comparison of weight is represented in Figure 2.

### 3.3. Body Mass Index (BMI)

**Figure 3:**
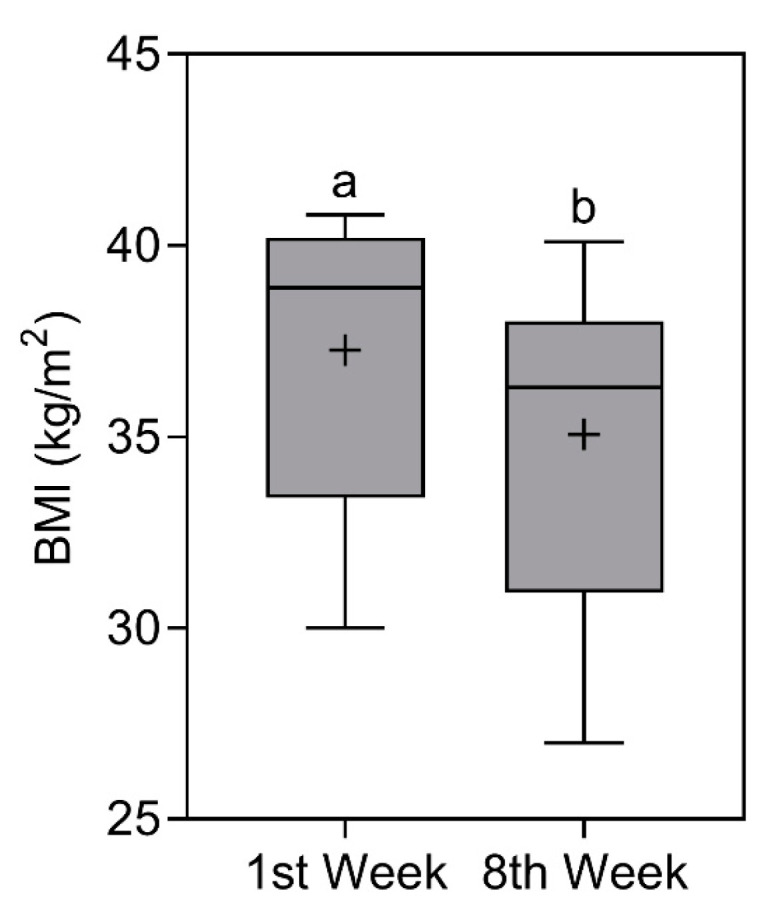
Body mass index (BMI) of study participants in the first and eighth week. Data presented as median (central boxplot line), mean (plus sign), interquartile range (lower box limit for Q1 and upper box limit for Q3), minimum (lower whisker), and maximum (upper whisker). Different superscript letters indicate a significant difference between the BMI observed in the first and eighth week (Wilcoxon test; *p* < 0.0001).

BMI also reduced significantly (*p* < 0.0001) from 38.9 (40.2–33.4) kg/m^2^ [$\bar{X}$ ± SD = 37.3 ± 3.9 kg/m^2^] in the first week to 36.3 (38–31) kg/m^2^ [$\bar{X}$ ± SD = 35.1 ± 3.9 kg/m^2^] in the eighth week. Even with reductions in the score, the classification remained the same, that is, “obesity II” (note: classification based on median values) [32]. The comparison of BMI is represented in Figure 3.

### 3.4. Waist Circumference

**Figure 4:**
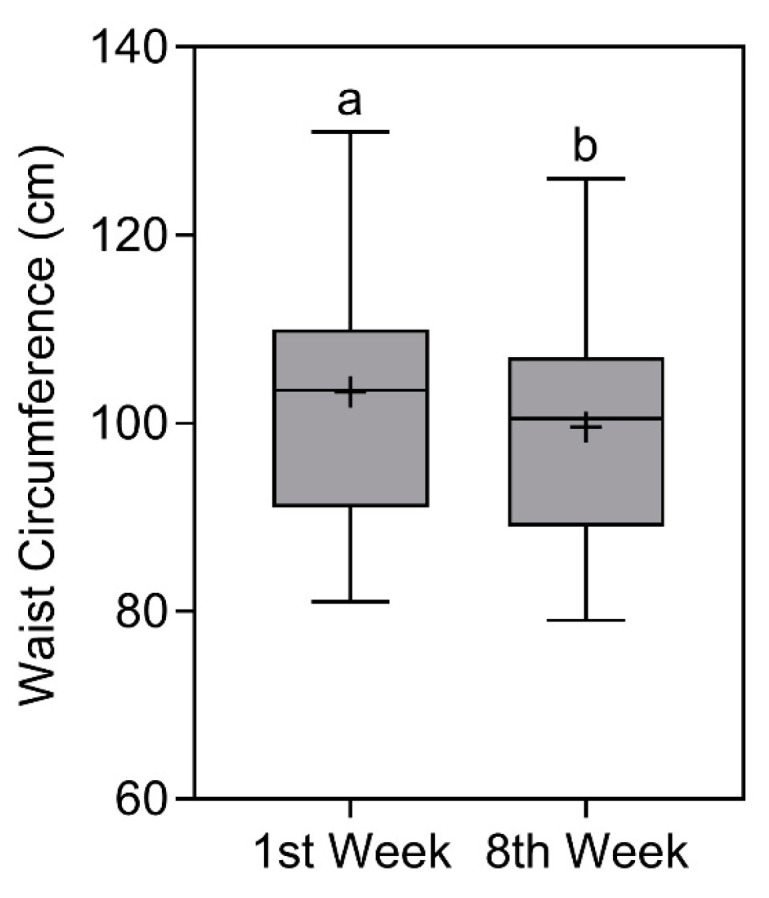
Waist circumference of study participants in the first and eighth week. Data presented as median (central boxplot line), mean (plus sign), interquartile range (lower box limit for Q1 and upper box limit for Q3), minimum (lower whisker), and maximum (upper whisker). Different superscript letters indicate a significant difference between the waist circumference observed in the first and eighth week (Wilcoxon test; *p* < 0.0001).

Waist circumference also reduced significantly over time (*p* < 0.0001). In the first week, the average value was 103.5 (110–91) cm [$\bar{X}$ ± SD = 103.3 ± 12.5 cm], classified as very high cardiovascular risk for men and women [32]. In the eighth week, the average value reduced to 100.5 (107–89) [$\bar{X}$ ± SD = 99.6 ± 11.6 cm], classified as very high cardiovascular risk for women and high cardiovascular risk for men [32] (note: classifications based on median values). The comparison of waist circumference is represented in Figure 4.

### 3.5. Body Shape Questionnaire (BSQ)

**Figure 5:**
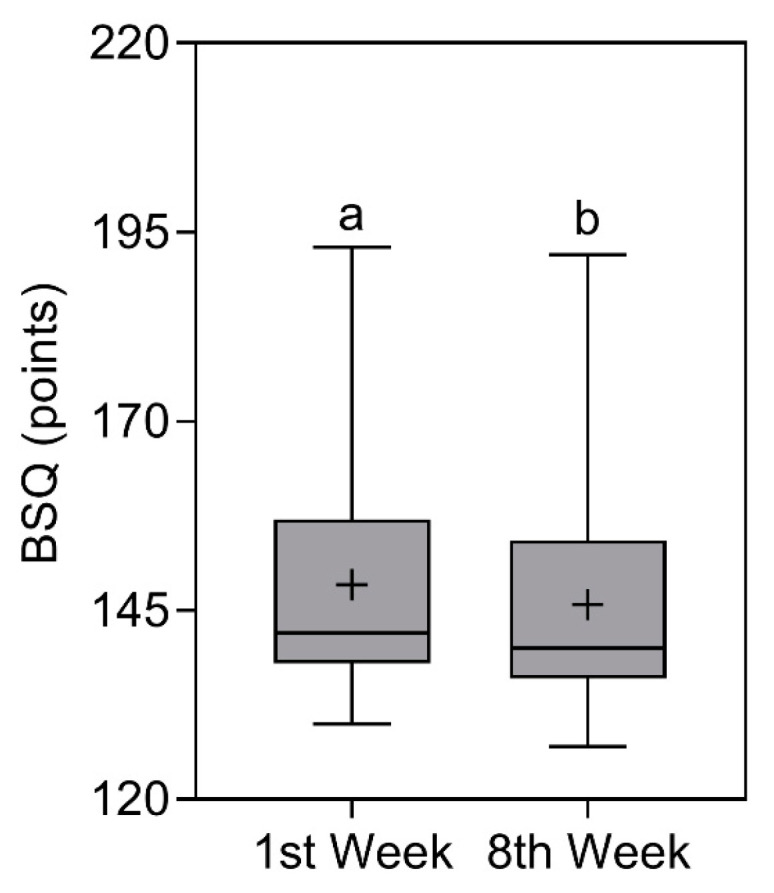
Body Shape Questionnaire (BSQ) results of study participants in the first and eighth week. Data presented as median (central boxplot line), mean (plus sign), interquartile range (lower box limit for Q1 and upper box limit for Q3), minimum (lower whisker), and maximum (upper whisker). Different superscript letters indicate a significant difference between the BSQ results observed in the first and eighth week (Wilcoxon test; *p* < 0.0001).

BSQ scores reduced significantly (*p* < 0.0001) from 142 (157–138) points [$\bar{X}$ ± SD = 148.4 ± 14.1 points] in the first week to 140 (154.3–136) points [$\bar{X}$ ± SD = 146 ± 14.3 points] in the eighth week. Even with reductions in the score, the classification remained the same, that is, “Dissatisfied with their body image” [27,28] (note: classification based on median values). The comparison of BSQ scores is represented in Figure 5.

### 3.6. Binge Eating Scale (BES)

**Figure 6:**
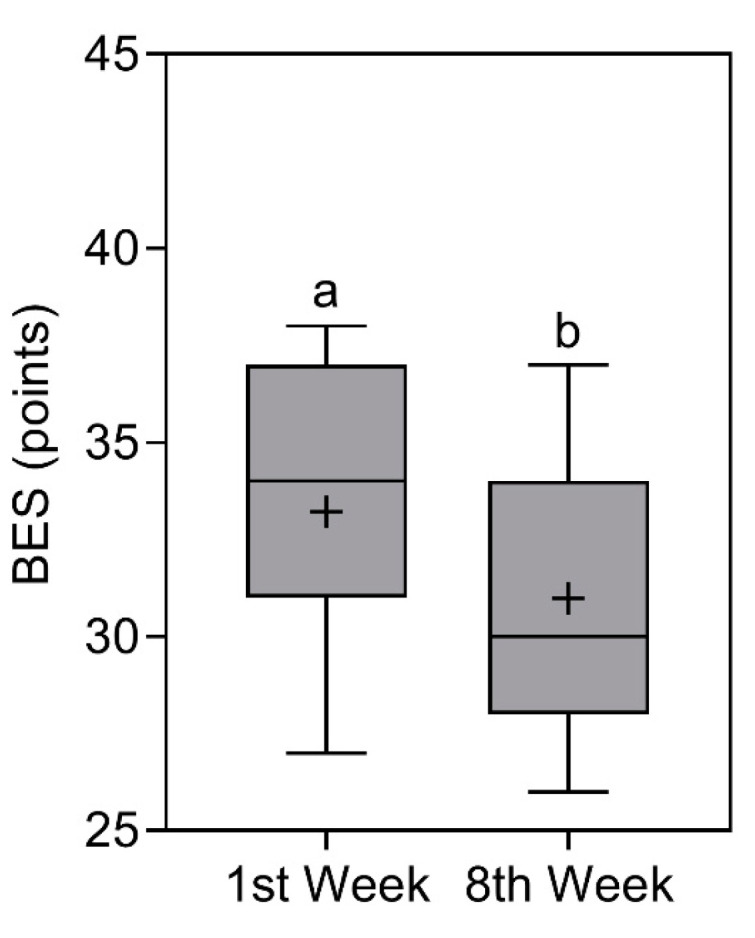
Binge eating scale (BES) of study participants in the first and eighth week. Data presented as median (central boxplot line), mean (plus sign), interquartile range (lower box limit for Q1 and upper box limit for Q3), minimum (lower whisker), and maximum (upper whisker). Different superscript letters indicate a significant difference between the BES observed in the first and eighth week (Wilcoxon test; *p* < 0.0001).

BES scores reduced significantly (*p* < 0.0001) from 34 (37–31) points [$\bar{X}$ ± SD = 33.2 ± 3.3 points] in the first week to 30 (34–28) [$\bar{X}$ ± SD = 31 ± 3.3 points] in the eighth week [29,30]. Even with reductions in the score, the classification remained the same, that is, “Severe binge eating” (note: classification based on median values). The comparison of BES scores is represented in Figure 6.

### 3.7. Binge Eating Episodes

**Figure 7:**
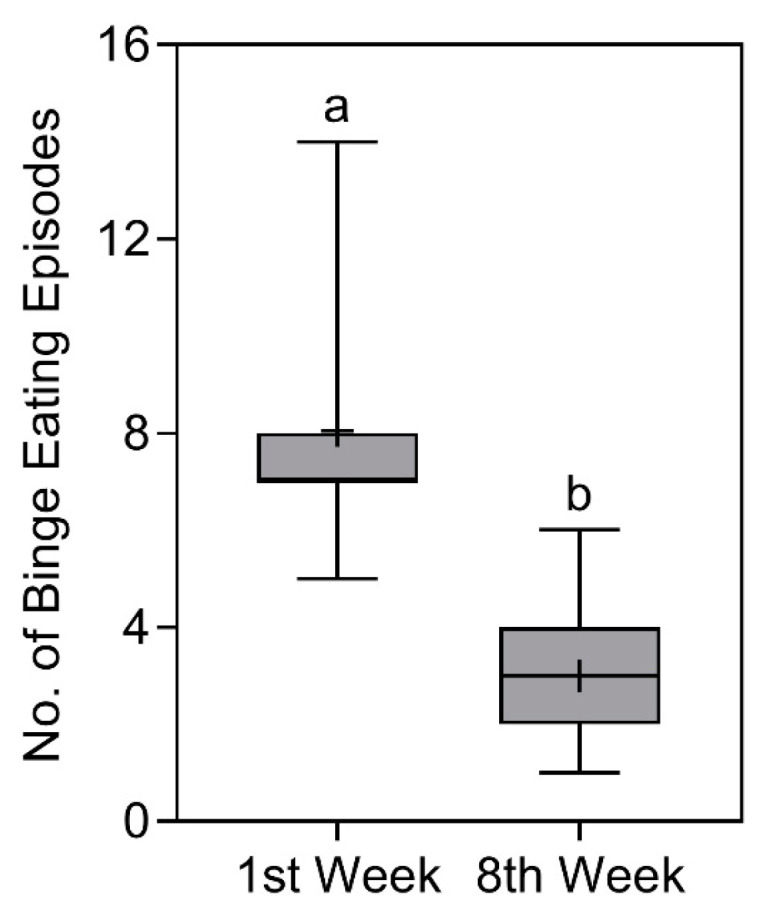
Number of binge eating episodes of study participants in the first and eighth week. Data presented as median (central boxplot line), mean (plus sign), interquartile range (lower box limit for Q1 and upper box limit for Q3), minimum (lower whisker), and maximum (upper whisker). Different superscript letters indicate a significant difference between the number of episodes observed in the first and eighth week (Wilcoxon test; *p* < 0.0001).

In the analyses of the number of binge eating episodes, a significant reduction (*p* < 0.0001) was detected from 7 (8–7) episodes [$\bar{X}$ ± SD = 8 ± 2.3 episodes] in the first week to 3 (4–2) episodes [$\bar{X}$ ± SD = 3 ± 1.4 episodes] in the eighth week. The comparison of the number of binge eating episodes is represented in Figure 7.

### 3.8. Quality of Life Scale—WHOQOL-BREF

**Figure 8:**
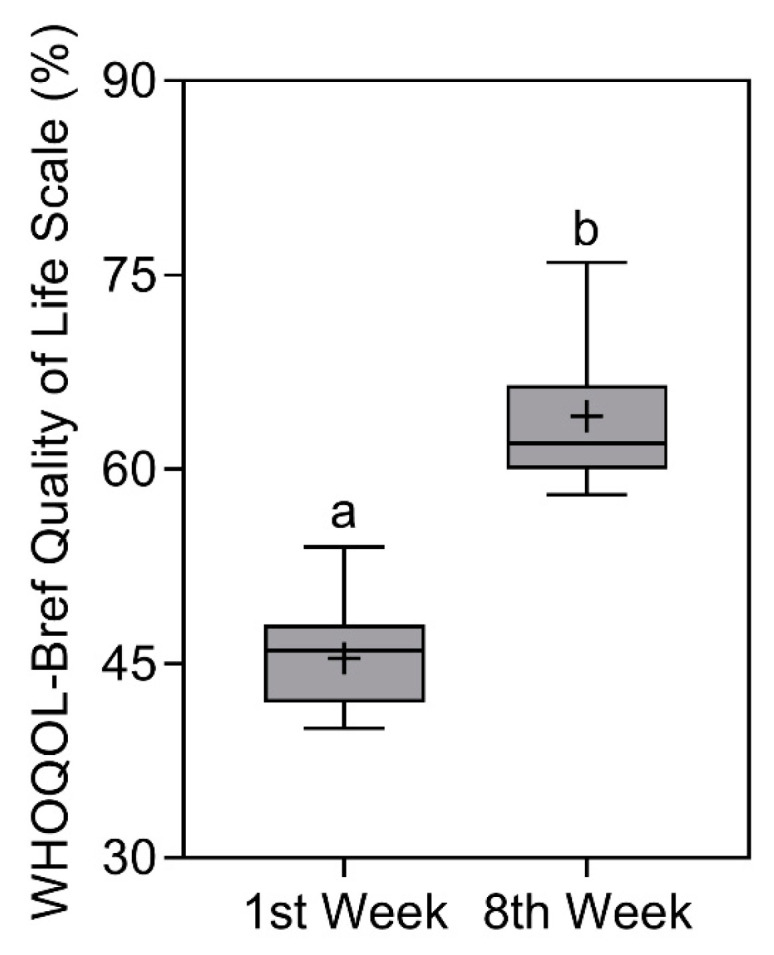
WHOQOL-Bref quality of life scale results of study participants in the first and eighth week. Data presented as median (central boxplot line), mean (plus sign), interquartile range (lower box limit for Q1 and upper box limit for Q3), minimum (lower whisker), and maximum (upper whisker). Different superscript letters indicate a significant difference between the results observed in the first and eighth week (Wilcoxon test; *p* < 0.0001).

Regarding the results of the WHOQOL-BREF Scale score [25,26], a significant increase in scores (*p* < 0.0001) was found from 46 (48–42) % [$\bar{X}$ ± SD = 45.4 ± 3.6%] in the first week to 62 (66.5–60) % [$\bar{X}$ ± SD = 64.1 ± 6%] in the eighth week. The comparison of the WHOQOL-BREF Scale is represented in Figure 8.

### 3.9. Additional Results for Qualitative and Quantitative Dietary Habits

In the patients’ food diaries, it could be seen that they were consuming fewer calories (approximately −350 kcal) than before the start of the intervention, and an increase in water intake was also found (*p* < 0.0001), as can be observed in Table 3. There was also a lower intake of ultra-processed foods with high caloric density (cakes, biscuits, chocolates, sweets, snacks, soft drinks, and fruit nectars), and a higher consumption of fruits, vegetables, legumes, rice, beans, lean proteins, whole grains, nuts, oilseeds, teas, spices, and aromatic herbs. The patients also reported that they were cooking and eating more meals at home (mainly rice and beans).

### 3.10. Follow-Up (By Telephone)

Two months after the intervention, the researchers contacted the participants (by telephone) to collect some information, since it was not possible to hold a new meeting. The follow-up happened in the second week of January, which is a month of vacation, and so many patients traveled in Brazil. Therefore, it was only possible to contact them by telephone. We aimed to assess whether the interventions promoted changes in or maintenance of eating habits in the long-term. Two questions were asked: “Did you have any changes in your weight after the intervention? Did you continue to be monitored by a health professional?”. Of the 82 participants, 71% reported that they managed to lose weight and continued to be monitored by a psychiatrist, psychologist, and nutritionist. However, 29% returned to their initial weight and said they had lost the motivation to continue treatments. The distance made data collection and information accuracy difficult, since there was no visual contact or analysis of anthropometric data.

## 4. Discussion

The positive effects on weight loss, BMI, and waist circumference may be a consequence of the fact that the nutritional intervention prioritized a behavioral approach that can help improve eating habits, optimizing the treatment of BED and obesity without necessarily changing or waiting for patients’ weight loss. Although no dietary prescription was performed, it was found that patients consumed fewer calories than previously. Furthermore, there was a lower intake of foods with high caloric density (ultra-processed), a greater intake of foods with low caloric density (fresh and minimally processed), more meals at home, and an increased hydration, which may have contributed to greater satiety and a negative energy balance. Most likely, ME contributed to improving food awareness, promoting a satietogenic effect, impacting TEV, and indirectly weight loss, since it encourages people to chew their food and eat calmly. According to the literature, chewing and eating slowly have a potential effect on the physiological and hormonal control of satiety signals (cholecystokinin and leptin) [13,14]. Otherwise, the literature indicates that anthropometric changes are not usually expected at the beginning of BED treatment [1], as the initial objective is to treat the psychological and behavioral aspects without expecting any weight loss [36]. The patient can often experience a slight initial weight gain due to the use of some types of psychiatric medications [1].

Additionally, body weight is an indicator that can affect the feelings and emotions of patients with obesity and BED, where dissatisfaction, distortion of body image, and a critical view of oneself are commonly observed [7]. In this regard, therapies based on acceptance and recognition of body image can be a good strategy to control both diseases in the long-term [37], remembering that the term “acceptance” does not mean “conforming” to one’s health status, but rather recognizing it and seeking treatment. Therefore, the patient has complete autonomy in wanting to take care of their health and recover their nutritional status [12,36].

As already mentioned, ME appears to be an alternative strategy for weight control, but unfortunately studies still present very contradictory results [20]. A recent randomized clinical trial analyzed 138 women who were divided into three weight loss groups. One of the strategies included a nutritional intervention with the practice of ME. The results demonstrated that there was weight loss in all groups, but with no statistical difference between them [38]. Apparently, the association of ME with calorie restriction did not promote significant weight loss and also presented several limitations [19,20].

On the other hand, a systematic review and meta-analysis investigated several trials involving ME and its real impact on weight loss. The results showed significant differences in weight loss, BMI, and waist circumference. Regardless, the authors concluded that ME and intuitive eating may be a practical and an inexpensive approach, but future research should aim to investigate their long-term effects and include a more heterogeneous study population [36]. In the present research, the selected sample appears to be heterogeneous (with female and male participants; any type of socioeconomic status, race, and age), which is another great strength, given the multiethnic characteristics and sociocultural diversity found in Brazil. In this sense, it can be noted that BED and obesity do not have a particular color, address, or socioeconomic status, just like many other types of chronic and psychiatric illnesses. They can affect everyone and are a problem to be addressed by all global health systems [5,39].

Another interesting finding of this study was the reduction in episodes of binge eating, which was most likely a consequence of the regularization of self-control, hunger, satiety signals, and reduction in excessive caloric intake. Loss of control is the main characteristic mediated by these episodes and, apparently, ME managed to affect the brain areas that can control decision making, such as the prefrontal cortex and the limbic system [12,13,40]. In this sense, interventions should focus on reducing binge eating episodes so that, in the long-term, they can indirectly contribute to reducing anthropometric data, improving eating habits, and increasing satisfaction with body image [14].

An improvement was also observed in the scores for the analyzed scales (BES, BSQ, and WHOQOL-BREF). Although the BES and BSQ classification did not change, there was a reduction in parameters concomitant with an increase in the WHOQOL-BREF score. This is a very strong point, as these patients face several problems with their body image that impact their quality of life and worsen their mental health [41]. Research involving the management of obesity and BED demonstrates their serious impact on the quality of life of patients who use healthcare services on a large scale, thus increasing healthcare costs [1].

Moreover, this study also observed an increase in the consumption of home-cooked meals. According to the “Dietary Guidelines for the Brazilian Population”, eating meals at home increases the patient’s relationship with food through the therapeutic act of cooking itself, promotes economic benefits, and raises the quality of the food consumed, as the patient knows exactly what is being ingested. An increase in the consumption of “rice and beans” was also observed. This typical dish is often eaten at lunch and dinner, accompanied by a salad and a protein source (animal or vegetable). “Rice and beans” are considered a Brazilian “staple dish”, consumed by everyone, with an excellent nutritional composition (qualitative and quantitative) and providing macro/micronutrients, fiber, and satiety throughout the day [23].

A recent systematic review and meta-analysis addressed different types of psychological and behavioral strategies, such as mindfulness and ME, to reduce emotional eating in patients who are overweight. The results showed good effectiveness in treating the individual’s relationship with food and the way they project their expectations regarding food [7]. Emotional eating is also observed in patients with BED and obesity [7,12] and is often triggered by a response to negative feelings (stress, upset, or furiousness) in search for temporary comfort by overconsuming fewer home-cooked meals and more ultra-processed food, with high energy density and palatability [13,42].

Due to the lack of evidence for the improvement of anthropometric parameters through the practice of ME, the results of this present investigation are considered an excellent start and suggest that the technique can be incorporated into nutritional interventions to improve, control, and sustain weight loss as well as patients’ eating habits. Furthermore, the program is simple and low-cost, making it suitable for all types of patients. However, despite the promising results, ME should not be seen as an easy miracle solution to an extremely complex treatment like that of obesity and BED, and needs to be studied in the future in a randomized clinical trial, especially in the long-term. In this sense, this technique can complement nutritional management while also optimizing treatment by the multidisciplinary team.

### 4.1. Follow-Up

Regarding the weight regain experienced by 29% of patients, even though they do not represent the largest portion of the sample, it gives an indication that it is necessary to understand the behavior of returning to baseline after the intervention. “Ups and downs” can be a part of the treatment of patients with obesity and BED. In this sense, future research needs to understand “why some patients are unable to continue the treatment alone?”. Probably because both diseases are complex, they do not have a total “cure”, but they can be controlled and require constant monitoring. This possibly shows the importance of a multidisciplinary team and monitoring by health professionals (nutritionist, psychologist, and psychiatrist) in the long-term [18]. Furthermore, the follow-up took place right after holidays and parties in Brazil (Christmas and New Year), where an increase in caloric intake and weight gain is already expected. However, 71% of patients maintained their weight loss. It is clear that some patients may have responded more to the intervention and ME technique than others. In this sense, it is noted that nutritional management by a multidisciplinary team is very important, improving health surveillance, care, and quality of life in different types of treatment and diseases [14,16].

### 4.2. Advantages, Strengths, and Perspectives

-This work provided important data on improvements in anthropometric measures, dietary composition, hydration, cooking at home, and quality of life parameters of the patients through the incorporation of a simple, cheap, and applicable technique in everyday life. This is extremely important in the public health context, especially for countries like Brazil, which is facing a financial crisis and is unable to allocate funds to the health sector for the treatment of mental illnesses or chronic non-communicable diseases, such as BED and obesity;

-The selected sample appears to be heterogeneous, which is another great strength of this study, given the multiethnic characteristics and great sociocultural diversity found in Brazil. In this sense, it is clear that BED and obesity are increasingly common in people of different types of socioeconomic status, gender, and age. Both diseases are a complex interaction of different factors for different people in different countries and cultures. A universal strategy for every person is never going to be a solution;

-This work presented interesting data on reducing binge eating episodes and calorie intake. In this sense, focusing attention on treatment to reduce episodes is an important point in initial treatment goals, as they provide a big energy intake in the short-term;

-This research also provides a good literary overview of particular details of the ME technique (introduction and abstract graph) and new implications for the treatment of obesity and BED. The results also are very promising, enabling new findings, elaboration of hypotheses, and development of new research in the future;

-This study also managed to control for possible factors that could impact the results, such as the use of medications and physical exercise, since the patients did not use them and were sedentary.

### 4.3. Limitations

-This was a short-term intervention (2 months). Extending the project can further optimize the results obtained in the long-term (6 to 12 months);

-It was not possible to include a control group, as the study was carried out at a nutritional clinic whose main objective is to recover the nutritional status of people with behavioral and clinical strategies. Moreover, all recruited patients who showed interest in participating in the study needed urgent treatment. In this way, the sample number was increased, aiming to mitigate this limitation;

-The sample also proved to be heterogeneous, which is another good indication of representation, especially in a multicultural country like Brazil. However, the inclusion of a control group is suggested, aiming to increase the level of evidence in future studies through the development of a randomized clinical trial;

-The sample size is “relatively” small. Nonetheless, it was very difficult to follow 82 patients for 8 weeks and also conduct a “cooking workshop” (fifth meeting) with all of them. The authors did a lot of work to execute this project;

-There are several types of questionnaires involving ME assessment in the literature, but at the time the research was being carried out, there was no very good translation and validation in Brazilian Portuguese. Furthermore, there is substantial variation between different ME measures. However, it is important to ensure that the selected measures sufficiently capture the characteristics of interest and care must also be taken when interpreting results, given the limitations of questionnaire-based ME measures. Although ME measures tend to show significant associations, for example, with weight and problematic eating behaviors, to date, sensitivity to change (useful for evaluating ME interventions) has not yet been properly established for the other measures. In future, it may be interesting to use or develop other questionnaires to verify the accuracy and improvement of ME throughout the intervention;

-The follow-up happened in the second week of January, which is a month of vacation, and many patients were traveling. Therefore, it was only possible to contact them by telephone. Distance made data collection and information accuracy difficult, since there was no visual contact or analysis of anthropometric data;

-In future studies, it may be interesting to evaluate body fat and muscle mass, since weight and BMI may have interferences and variations, depending on each patient;

-It would also be interesting to apply a satisfaction survey and suggestions to the 82 individuals who participated in the eight meetings, aiming to improve the techniques and protocols developed.

## 5. Conclusions

ME may reduce the number of binge eating episodes, improve anthropometric data, body image dissatisfaction, quality of life, and eating habits in patients with obesity and BED in the short-term. Furthermore, this technique appears to be inexpensive and has also proven to be effective in a heterogeneous sample. In this way, this therapy can help to recover the nutritional status of patients, complement nutritional management, and optimize treatment conducted by multidisciplinary team. However, an extension of the project will be necessary to analyze the impact of the intervention in the long-term.

## 6. Highlights—Take-Home Message

- There is still little evidence of the practice of mindful eating (ME) for the improvement of anthropometric data (weight, BMI, and waist circumference) in patients with obesity and binge eating disorder (BED);
- This study provides an update on the development of nutritional interventions that incorporate the practice of ME to optimize the complex treatment of this public health concern;
- This research study combined nutrition with psychology to understand what happens on the physiological and emotional planes in patients who have obesity and BED;
- It is possible to promote nutritional interventions that include qualitative subjective nutritional management without necessarily being a standard dietary prescription and that also present good results;
- ME should not be seen as a miracle treatment, but rather a strategy to complement nutritional management;
- Obesity and BED are a complex interaction of different factors for different people in different countries and cultures. They do not have a specific color, address, or socioeconomic status. They can affect anyone and deserve the attention of all health systems in the world. One universal strategy for every person is never going to be the solution.

## Figures and Tables

**Table 1:** Summary of Nutritional Interventions.

Individual Weekly Meetings	Goals	Procedures
1st meeting: interview, evaluation, and data collection	(1) Get to know and approach the patients. (2) Evaluate clinical and sociodemographic data, level of quality of life, risk of BED, and dissatisfaction with body image at the 1st meeting. (3) Teach and apply ME techniques using favorite food dynamics.	(1) Informal introductory chat to get to know the patients and learn about all the points and dynamics that will be developed in the intervention. (2) Application of protocols: “Clinical and Sociodemographic Protocol” [24]; “Quality of Life Scale WHOQOL-BREF” [25,26]; “Body Shape Questionnaire (BSQ)” [27,28]; “Binge Eating Scale (BES)” [29,30]. (3) Learn and develop the habit of enjoying food; identify signs of hunger and satiety; develop the act of paying attention to food; eat calmly and without rushing; control breathing to reduce anxiety before eating. All techniques were applied while patients ate their favorite foods that they brought to the meeting.
2nd meeting: nutritional intervention	(1) Provide feedback on questionnaire results to participants. (2) Develop a “food diary” to increase food awareness and encourage autonomy in food intake. (3) Make a qualitative individual healthy eating plan, without prescribing calories, carbohydrates, proteins, fiber, and fats. (4) Apply ME techniques using favorite food dynamics.	(1) Discussion of the questionnaires, aiming to clarify the diagnosis, results, scores obtained, and possible treatments for obesity and BED. (2) Patients were encouraged to write down all the foods they ate, quantities, times, and other information. (3) Encouragement to eat more natural, minimally processed food; eat fewer ultra-processed foods; drink more water. (4) Develop the habit of enjoying food; identify signs of hunger and satiety; develop the act of paying attention to food; eat calmly and without rushing; control breathing to reduce anxiety before eating. All techniques were applied while patients ate their favorite foods that they brought to the meeting.
3rd meeting: nutritional intervention	(1) Explanation of the history, theory, practice, and studies involving ME. (2) Apply ME techniques using favorite food dynamics.	(1) Informative chat with patients, seeking to teach and understand what patients knew about the technique. (2) Develop the habit of enjoying food; identify signs of hunger and satiety; develop the act of paying attention to food; eat calmly and without rushing; control breathing to reduce anxiety before eating. All techniques were applied while patients ate their favorite foods that they brought to the meeting.
4th meeting: nutritional intervention	(1) Clarify the myths and truths on healthy eating. (2) Apply ME techniques using favorite food dynamics.	(1) Clarification of food myths and truths published in the media: carbohydrates are not the villain; gluten and lactose do not need to be excluded from your diet; you do not need to starve to lose weight; intermittent fasting is not the best nutritional strategy; eating carbohydrates at night does not make you gain weight; and others. (2) Develop the habit of enjoying food; identify signs of hunger and satiety; develop the act of paying attention to food; eat calmly and without rushing; control breathing to reduce anxiety before eating. All techniques were applied while patients ate their favorite foods that they brought to the meeting.
5th meeting: nutritional intervention	(1) Encourage participants’ culinary habits through culinary workshops and food tasting. The recipes were developed in the clinic’s kitchen. (2) Apply ME techniques using favorite food dynamics.	(1) Activity in the kitchen and active participation in cooking traditional Brazilian recipes: salads; basic dishes (rice, beans, protein sources, and whole grains); typical dishes (“feijoada”, fish “moqueca”, shrimp “bobo”, and “galinhada”); refreshing juices; fruit-flavored water. (2) Develop the habit of enjoying food; identify signs of hunger and satiety; develop the act of paying attention to food; eat calmly and without rushing; control breathing to reduce anxiety before eating. All techniques were applied while patients ate their favorite foods that they brought to the meeting.
6th meeting: nutritional intervention	(1) Evaluate the relationship of patients with food through blindfolded sensory analysis. (2) Apply ME techniques using favorite food dynamics.	(1) The participants’ eyes were blindfolded and they were presented with some foods (fruits, vegetables, spices, and herbs). Patients could only use touch, smell, and taste to elucidate them. (2) Develop the habit of enjoying food; identify signs of hunger and satiety; develop the act of paying attention to food; eat calmly and without rushing; control breathing to reduce anxiety before eating. All techniques were applied while patients ate their favorite foods that they brought to the meeting.
7th meeting: nutritional intervention	(1) Talk about eating disorders and eating behavior. Apply measures to channel some symptoms caused by BED. (2) Apply ME techniques using favorite food dynamics.	(1) Discover patients’ opinions about the symptoms of eating disorders in social situations. (2) Develop the habit of enjoying food; identify signs of hunger and satiety; develop the act of paying attention to food; eat calmly and without rushing; control breathing to reduce anxiety before eating. All techniques were applied while patients ate their favorite foods that they brought to the meeting.
8th meeting: final evaluation and data collection	(1) Discuss and complete interventions through mindfulness and ME videos. (2) Evaluate clinical and sociodemographic data, quality of life, risk of BED, and dissatisfaction with body image at the 8th meeting. (3) Apply ME techniques using favorite food dynamics.	(1) Patient feedback on nutritional interventions and completion of activities with audiovisual dynamics. (2) Reapplication of protocols: “Clinical and Sociodemographic Protocol” [24]; “Quality of Life Scale WHOQOL-BREF” [25,26]; “Body Shape Questionnaire (BSQ)” [27,28]; “Binge Eating Scale (BES)” [29,30]. (3) Develop the habit of enjoying food; Identify signs of hunger and satiety; Develop the act of paying attention to your food; Eat calmly and without rushing; Control your breathing to reduce anxiety before eating. All techniques were applied while patients ate their favorite foods that they brought to the meeting.

**Table 2:** Sociodemographic data of the sample.

Variables	Total Sample Size *(n* = 82)	Total of the Individuals (%)
**Sex**		
Female	*n* = 47	57.3%
Male	*n* = 35	42.7%
**Economic Class**		
A	*n* = 9	11.0%
B	*n* = 23	28.0%
C	*n* = 33	40.2%
D	*n* = 8	9.8%
E	*n* = 9	11.0%
**Race**		
White	*n* = 16	19.5%
Black	*n* = 18	22.0%
Brown	*n* = 14	17.1%
Indigenous	*n* = 12	14.6%
Yellow	*n* = 22	26.8%
**Age**		
From 37 years to 56 years	*n* = 82	N/A
Md ± IQR = 48.5 (51–43)		
$\bar{X}$ ± SD = 47.5 ± 4.8		

Observations: Class A (those earning more than 20 minimum wages), Class B (from 10 to 20 minimum wages), Class C (from 4 to 10 minimum wages), Class D (from 2 to 4 minimum wages), and Class E (receives up to 2 minimum wages). N/A= not applicable. Age is represented as a descriptive range (minimum and maximum), below are median ± interquartile range (IQR) (Md ± IQR), and additionally mean ± standard deviation (SD) values ($\bar{X}$ ± SD).

**Table 3:** Total energy value (VET) and daily water intake (L) results of study participants in the first and eighth week.

Variables	1st Week	8th Week	*p*
**TEV (Kcal) (median ± IQR)**	2552 (2555–2550) * [$\bar{X}$ ± SD = 2552 ± 2.5]	2202 (2204–2201) * [$\bar{X}$ ± SD = 2203 ± 2.4]	<0.0001
**Water Intake (L) (median ± IQR)**	1.5 (1.6–1.5) * [$\bar{X}$ ± SD = 1.5 ± 0.2]	3.1 (3.5–3.1) * [$\bar{X}$ ± SD = 3.2± 0.2]	<0.0001

Data presented as median ± interquartile range (IQR). (IQR = Q3 − Q1). An asterisk (*) indicates a significant difference of values observed between the 1st and 8th weeks (Wilcoxon test; *p* < 0.0001). For curiosity, mean ± standard deviation (SD) values (represented by the symbol $\bar{X}$ ± SD) are also indicated below the median ± interquartile range (IQR).

## Data Availability

Study data were collected and managed using REDCap 14.0.9 electronic data capture tools hosted at REDCap—FUNFARME/FAMERP (from State Faculty of Medicine) [10,11]. The data presented in this study are available on request from the corresponding author. The data are not publicly available due to privacy.
